# Nondrug Intervention for Opportunistic Infections in Individuals With Hematological Malignancy: Systematic Review

**DOI:** 10.2196/43969

**Published:** 2023-03-31

**Authors:** Nor Asiah Muhamad, Nur Hasnah Ma'amor, Normi Mustapha, Fatin Norhasny Leman, Izzah Athirah Rosli, Marilyn Umar, Tahir Aris, Nai Ming Lai

**Affiliations:** 1 Sector for Evidence-based Healthcare National Institutes of Health Ministry of Health Shah Alam Malaysia; 2 Faculty Science and Technology Open University Kuala Lumpur Malaysia; 3 Non-Communicable Disease Section, Sarawak State Health Department Ministry of Health Sarawak Malaysia; 4 Director's Office, Institutes for Medical Research National Institutes of Health Ministry of Health Shah Alam Malaysia; 5 School of Medicine, Faculty of Health & Medical Sciences Taylor's University Subang Jaya Malaysia

**Keywords:** nondrug, intervention, opportunistic infection, hematological malignancies

## Abstract

**Background:**

Hematological malignancies disturb the blood, lymph nodes, and bone marrow. Taking medications for treating opportunistic infections (OIs) in these individuals may enhance the risk of medication interaction as well as adverse drug reactions.

**Objective:**

This review aims to evaluate the effectiveness of nondrug interventions in reducing OIs among patients with hematological cancers.

**Methods:**

The PubMed, CENTRAL (Cochrane Central Register of Controlled Trials), and Embase databases were searched on December 26, 2022, for all randomized controlled trials (RCTs). The primary endpoint was OIs. The quality of included studies was assessed by the Cochrane Risk-of-Bias tool.

**Results:**

A total of 6 studies were included in this review with 4 interventions: (1) types of mouthwash received, (2) presence of coating on central venous catheters (CVCs), (3) use of well-fitted masks, and (4) types of diet consumed. The results were presented in 8 different comparisons: (1) chlorhexidine-nystatin versus saline mouth rinse, (2) chlorhexidine versus saline mouth rinse, (3) nystatin versus saline mouth rinse, (4) chlorhexidine silver sulfadiazine–coated CVCs versus uncoated catheters, (5) well-fitted masks versus no mask, (6) amine fluoride-stannous fluoride versus sodium fluoride mouthwash, (7) low-bacterial diet versus standard hospital diet, and (8) herbal versus placebo mouthwash. No clear differences were reported in any of the outcomes examined in the first 3 comparisons. There were also no clear differences in the rate of catheter-related bloodstream infection or insertion site infection between the use of chlorhexidine silver sulfadiazine–coated CVCs versus uncoated catheters in the patients. Further, no significant differences were seen between patients who used a well-fitted mask and those without a mask in the incidence of OI. The all-cause mortality and mortality due to OI were similar between the 2 groups. There was no clear difference in all-cause mortality, although common adverse effects were reported in patients who used sodium fluoride mouthwash compared with those using amine fluoride-stannous fluoride mouthwash. There was no evidence of any difference in the incidence of possible invasive aspergillosis or candidemia between patients who consumed a low-bacterial diet and a standard diet. For the last comparison, no significant difference was seen between patients who received herbal and placebo mouthwash.

**Conclusions:**

Very limited evidence was available to measure the effectiveness of nondrug interventions in hematological cancers. The effectiveness of the interventions included in this review needs to be evaluated further in high-quality RCTs in a dedicated setting among patients with hematological malignancies.

**Trial Registration:**

PROSPERO International Prospective Register of Systematic Reviews CRD42020169186; https://www.crd.york.ac.uk/PROSPERO/display_record.php?RecordID=169186

## Introduction

### Background

Hematological cancer or malignancies are cancers of the blood, bone marrow, and lymph nodes that come from either lymphoid cell lines or myeloid. Megakaryocytes, macrophages, granulocytes, mast cells, and erythrocytes are produced by the myeloid cell line. The myeloid cell line is responsible for acute and myelodysplastic syndromes, myeloproliferative disorders, and chronic myelogenous leukemia [1]. Other cells such as plasma, T and B cells, as well as natural killer cells or large granular lymphocytes are produced by the lymphoid cell line. This lymphoid cell line is responsible for lymphomas, lymphocytic leukemia, and myelomas [1]. In the United States and United Kingdom, 16 (13.6%) leukemia cases are reported per 100,000 people [2,3]. The clinical course and prognosis vary as it may depend on the existence or the type of genetic mutation itself. Chemotherapy, radiation, immunotherapy, and bone marrow transplantation are examples of active treatment options.

A healthy individual with a strong immune system is usually resistant to opportunistic infections (OIs) caused by pathogens such as protozoa, fungi, viruses, or bacteria [1]. However, if the immune system is weakened, the pathogens have a better chance of infecting the individual. Cancer therapy to treat hematological cancer can weaken a patient’s immune system, making them vulnerable to OIs. OIs affect 5%-60% of individuals with hematological malignancies [4-7], with the mortality rate ranging between 15% and 41% [5]. Broad-spectrum intravenous antimicrobials are used to treat OIs, which are first used empirically and then adapted to the patient’s specific needs [8]. Viruses causing the OIs can be treated with antiviral therapies.

Most individuals with hematological cancers are more susceptible to OIs due to both immunological impairment of cancer therapies and disturbances caused by the disease itself [9,10]. In a situation where patients with cancer become neutropenic, they are isolated as a precautionary measure to prevent OIs, while the length of stay in the isolation room varies depending on their medical condition. This may cause wide range of psychological burdens such as depression, anxiety, and stress as a result of staying in the isolation room [11]. It has been reported that the psychological well-being of patients with cancer influences their treatment response and long-term prognosis [12-14]. Nondrug therapies such as isolation have been used to reduce the OIs. The relation between OIs and cancer treatment is bidirectional. Treatment for various types of cancers causes immunosuppression and that may cause OIs among patients with cancer. Treatment knowledge, understanding, and adherence will improve the overall prognosis [15,16]. Therefore, the objective of this review is to evaluate the efficiency of nondrug interventions in preventing OIs in individuals with hematological cancers. As a result, this study was performed to assess the safety and efficacy of nondrug interventions for the prevention of OIs in individuals with hematological cancer or malignancies.

### Types of Nondrug Interventions

There are three major types of nondrug interventions. The first type of intervention is the barrier method to prevent potential transmission such as wearing protective equipment or gloves, cleaning of bed sheets and clothes, and the use of masks. The second type of intervention is complete elimination of the causal agents such as fumigation on a regular basis, regular cleaning of potential microbe-harboring goods including toys and carpets, elimination of houseplants that are likely to be a reservoir for microorganisms, and the use of mouthwash as a personal hygiene modification [17]. The third type of intervention is the physical method such as applying positive pressure (controls the air quality in-flow) or using a high-efficiency particulate absorption (HEPA) filtered room to improve the hospital environment.

In patients with neutropenia, primary infections can be a result of mild injuries caused by venous and vascular catheters, which can spread through the bloodstream and eventually result in soft tissue and skin infection [18]. Neutropenic diet is also a part of the barrier method to reduce the risk of infection. It consists of a low-bacteria diet, such as meals cooked thoroughly or with boiled water. Despite its widespread use, the effectiveness of a neutropenic diet in patients undergoing chemotherapy remains debatable [19-21].

Physical methods such as using an HEPA filter may help prevent contact with pathogens that exist in soil or plants and can reduce OIs in vulnerable individuals [22-26].

Personal hygiene modifications, such as the use of mouthwash [27], chlorhexidine baths [28], and frequent cleaning of surfaces, may decrease the microorganisms and completely eliminate them [29].

### How the Intervention Might Work

Targeted nondrug interventions to treat OIs act in 3 ways: removing the cause of illness, reducing the contact with infectious agents, and decreasing the risk of microorganism invasion.

The use of personal protective equipment (PPE) and HEPA filters are examples of barrier methods to reduce one’s exposure to an infective agent. HEPA filters are reported to eliminate 99.97% of particles with a diameter over 0.3 m, which includes most microorganisms [30]. The use of PPE such as surgical and N95 masks has demonstrated beneficial effect in reducing the transmission of influenza virus [31,32]. Hand hygiene has been shown to prevent the transfer of harmful microorganisms, such as central line–acquired bloodstream infections and methicillin-resistant *Staphylococcus aureus* [33,34]. The use of gloves may limit the quantity of microbes’ transmission through skin contact. Alcohol-based antiseptics have been suggested to reduce pathogen transmission by denaturing the microorganisms’ proteins [1].

### Justification for This Review

OIs contribute to mortality and morbidity in individuals with hematological cancer. In these patients, nondrug interventions, such as the use of PPE and modifying the environment, are commonly used, as they may lessen the need for medication prophylaxis and therapy; however, they are not without hazards and expenses. As a result, synthesizing the existing evidence on the safety and efficacy of these interventions is critical. In that regard, this study was performed to assess the safety and efficacy of nondrug interventions for OI prevention in individuals with hematological cancer or malignancies.

## Methods

### Overview

We conducted a systematic review by following the PRISMA (Preferred Reporting Items for Systematic Reviews and Meta-Analyses) guidelines [35]. The PRISMA checklist was used while writing this report [36].

### Eligibility Criteria

For this review, we followed the PICOS mnemonic, where “P” represents adult patients with hematological cancer, and we included all individuals or patients regardless of the stage of disease, type of hematological cancer or malignancy, morbidity, and the received treatment modality; “I” is any nondrug intervention included either alone or in combination with other therapies such as alteration of patient/caregiver behavior, alteration of the home-based environment, and hospital-based environmental control measures. We also included other interventions defined by the study authors as nondrug intervention. The control “C” group is defined as individuals or patients who did not receive the nondrug intervention, or those who obtained prophylactic pharmacological medications or therapies. The outcomes “O” are defined as either OIs or bacterial OIs, mortality due to OIs, all-cause mortality, hospitalization duration in days, chemotherapy interruption (number of episodes or duration of interruption), quality of life, and adverse effects related to the intervention. The included study design “S” is all randomized controlled trials (RCTs) or cluster RCTs that are published in full texts or abstracts. We excluded studies when the intervention contained pharmacological measures or any studies with the crossover design due to concerns of the “carryover” effects.

### Search Methods for Identification of Studies

A comprehensive systematic literature search was performed on various electronic databases, Embase, Cochrane Central Register of Controlled Trials (CENTRAL), WHO (World Health Organization) International Clinical Trials Registry Platform (ICTRP), and PubMed, to identify relevant studies from inception to December 2022. A strategy search for PubMed was established and modified for use in the other databases. During the search, keywords and equivalent MeSH (Medical Subject Headings) phrases were combined when applicable, with no language or publication year restrictions. The search strategies for MEDLINE are presented in Multimedia Appendix 1 and CENTRAL in Multimedia Appendix 2. We also searched the abstract records from the following conferences organized by societies that are related to blood cancer or malignancies: The European Society for Medical Oncology Annual Congress, The European Hematology Association (EHA) Conference, American Association for Cancer Research and American Society of Clinical Oncology Conference, American Society of Hematology Meeting, and Virginia Association of Hematologists and Oncologists (VAHO) Spring Membership Conference. We also searched the following databases for ongoing studies: metaRegister of Controlled Trials [37], International Clinical Trials Registry Platform [38], and ClinicalTrials.gov [39]. During the searches, there was no limitation or restriction on the language of the article.

### Strategy for Data Collection and Analysis

#### Selection of Studies

**Figure 1:**
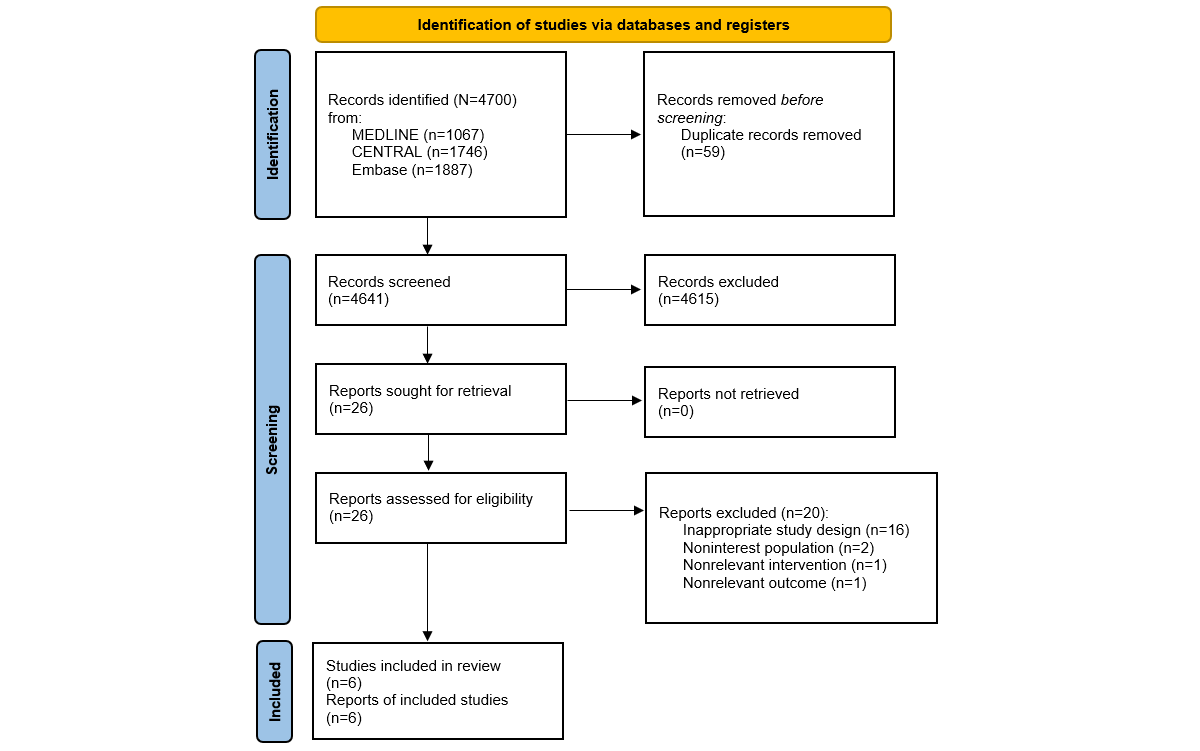
PRISMA flow diagram of studies selection. PRISMA: Preferred Reporting Items for Systematic Reviews and Meta-Analyses.

Two authors (NAM and NHM) independently screened all the study titles and abstracts and excluded studies that were not eligible. We resolved any discrepancies through discussion or by consultation with the third review author (TA). We followed the Cochrane Handbook for Systematic Reviews of Interventions [40] for reporting biases. We retrieved the full-text study reports/publications and 2 other review authors (NM and FNL) independently screened the full text to identify studies for inclusion, as well as identifying and recording reasons for exclusion of the ineligible studies. We resolved any disagreement through discussion or consulted a third review author (NML) to make the final judgment. We identified and excluded duplicates and collated multiple reports of the same study such that each study rather than each report is the unit of interest in the review. We recorded the selection process in sufficient detail to complete a PRISMA flow diagram (Figure 1) and tabulated the characteristics of the included and excluded studies [35].

#### Data Extraction and Management

Two authors (NAM and IAR) independently extracted the data and completed the data extraction using a standardized data collection form for study characteristics and outcome data. The form contained information on methods, total participants, interventions, comparisons, outcomes, and study design. We resolved disagreements by consensus.

#### Assessment of the Risk of Bias in Included Studies

**Figure 2:**
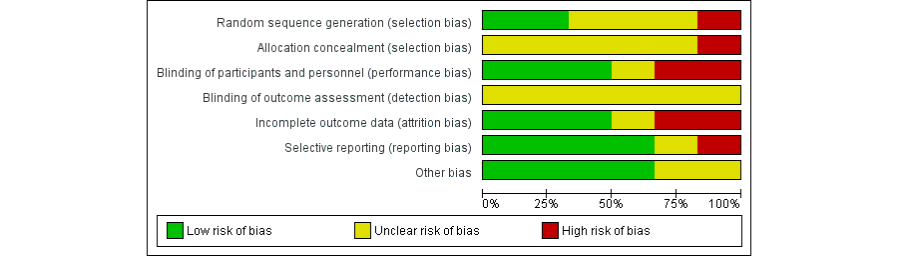
Risk of bias graph on review authors’ judgments about each risk of bias item presented as percentages across all included studies.

**Figure 3:**
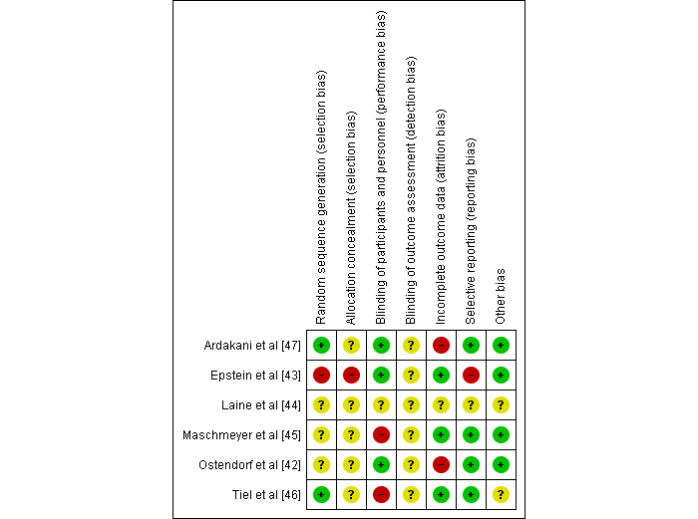
Risk of bias summary on review authors’ judgments about each risk of bias item for each included study.

Two review authors (NAM and MU) independently assessed the quality of the included studies using the Cochrane Risk-of-Bias Tool [40]. The risk of bias is assessed using the following 6 domains: (1) random sequence generation, (2) allocation concealment, (3) blinding of individuals or participants and personnel, (4) blinding of the outcome assessment, (5) incomplete data outcome, and (6) selective reporting and other bias. We summarized the risk-of-bias judgments for each of the domains listed in the “risk-of-bias” table included in Multimedia Appendix 3 for the 6 included studies and present our overall assessment of the risk of bias using a “risk-of-bias” graph (Figure 2) and “risk-of-bias” summary (Figure 3). Any disagreement among the review authors was resolved by discussion to achieve a consensus.

#### Data Synthesis

Dichotomous data were determined as risk ratio (RR) and reported with the 95% CI, whereas continuous data were observed as mean difference (MD) and the respective 95% CI [40]. Heterogeneity of treatment effects was measured using the *χ*^2^ test and the degree of heterogeneity was assessed using the *I*^2^ statistic, with the value of 75% or higher indicating substantial heterogeneity [40]. Two authors (NAM and NM) performed data analysis using Review Manager version 5.4 [41]. Meta-analysis was not possible because we could not include more than 1 study that provided usable data in a single comparison.

We created “summary of findings” (SOF) tables using the software GRADEpro (McMaster University and Evidence Prime Inc.; Tables S1-S8 in Multimedia Appendix 4). In the SOF tables, we included the following major outcomes, regardless of whether the outcome data were available [40]: (1) OI, as reported variously by the study authors; (2) all-cause death or mortality; (3) death or mortality that is associated with OI; (4) duration of hospitalization; (5) quality of life; and (6) adverse effects (either chemotherapy associated or attributable to the intervention examined).

In a comparison that evaluated chlorhexidine silver sulfadiazine–coated central venous catheters (CVCs) versus noncoated catheters [42], the major outcome reported was that the catheters were associated with various infections (catheter colonization, catheter-associated bloodstream infection, and insertion site infection). For this review, we have grouped these outcomes together with our predefined outcome of “OI” and have displayed these outcomes in the SOF tables.

## Results

### Findings From The Search Strategies

A total of 4700 records (1746 records from CENTRAL [Cochrane Central Register of Controlled Trials], 1067 records from MEDLINE, and 1887 records from Embase) were successfully retrieved. No additional records were identified through other sources, such as online conference archives and clinical trial registries. After removing duplicates, 4641 records remained. Subsequently, 4615 records were excluded. We obtained 26 records to be assessed for eligibility. Of these, 20 articles were excluded. Ultimately, 6 eligible articles or studies were included. The PRISMA flow diagram of the selection of studies is depicted in Figure 1.

### Included Studies

The 6 included studies were published as full papers [42-47]. Of these 6 studies, 2 were performed in Germany [42,45], 1 was conducted in Canada [43], 1 in Finland [44], 1 in The Netherlands [46], and 1 in Iran [47]. Table 1 describes in detail the characteristics of the studies included in this review.

**Table 1:** Characteristics of the included articles or studies.

Study	Study design	Participants	Interventions	Findings or outcomes	Notes
Epstein et al [43]	A single-center 4-arm randomized controlled trial conducted in Canada (published in 1992)	A total of 99 adult (>18 years old) patients diagnosed with leukemia with severe neutropenia. All patients were treated with chemotherapy with or without bone marrow transplantation. No exclusion criteria were stated.	Chlorhexidine, chlorhexidine + nystatin, nystatin, and saline rinse groups.	Chemotherapy-related oral complications such as oral mucositis, ulceration, gingivitis, and dental plaque.	This study was conducted for eligible individuals or patients who were admitted under the Leukemia/Bone Marrow Transplantation Service, Vancouver General Hospital, Canada. The study obtained the ethical review board approval of the Vancouver General Hospital. There was no conflicts of interest reported between the authors.
Laine et al [44]	A single-center randomized controlled trial conducted in Finland (study period was not stated)	A total of 76 adult patients who had been diagnosed with Hodgkin disease/non-Hodgkin lymphoma, which was confirmed by histological analysis. These patients received chemotherapy with curative intent. Their estimated life expectancy was <1 year. Patients were eligible if they did not have other concomitant disease, were receiving cancer therapy medication only, and with a Karnofsky Performance Status score of ≥60. Exclusion criteria were not explicitly stated.	Using a mouthwash containing 0.025% fluoride known as amine fluoride-stannous fluoride or 0.05% of sodium fluoride solution.	All-cause mortality and adverse effects such as stinging pain near the mouth, staining teeth, nausea, bad taste, and combined adverse effects, as well as salivary microbial count and salivary secretion rate.	This study was partly supported by the pharmaceutical industry (Gaba International Ltd., Basle, Switzerland) and partly by the Linda Gadd Foundation of the Finska Lakaresallskapet.
Maschmeyer et al [45]	A prospective randomized study conducted in Germany from February 2004 to October 2005	A total of 80 hospitalized patients (>18 years old) who had received either chemotherapy/intensive myelosuppressive therapy or allogeneic stem cell transplantation for acute leukemia. Exclusion criteria were clearly stated.	Patients received the standard or routine prophylaxis with or without a well-fitted FFP2^a^ mask.	The primary outcome was the occurrence of a possible, probable, or proven aspergillosis. Secondary outcomes were tolerability, patient compliance with wearing masks and other procedures related to infection prevention, mortality, administration of systemic antifungal agents for empirical or targeted treatment of invasive mycoses, and diagnosis of fungal infection within 2 weeks after the study.	The protocol of the study was approved by the Human Ethics Committee of the Charité University of Berlin, Germany. This study was performed in collaboration with 3M Germany, which provided the masks for free.
Ostendorf et al [42]	A single-center randomized controlled trial conducted in Germany	Individuals or patients with hematological cancer or malignancy. These patients needed or were on a CVC^b^ for a minimum of 7 days. A total of 184 CVCs were evaluated. The exclusion criteria were not mentioned in this study.	Chlorhexidine silver sulfadiazine–impregnated CVC versus nonimpregnated CVC.	Catheter colonization (mentioned as “catheter-related bacteremia”), mortality rate, and catheter-associated local infection.	This study received funding from the industrial partners (eg, from the distributor and manufacturer of catheter).
Tiel et al [46]	A randomized and controlled pilot study conducted in The Netherlands from February to December 2003	About 20 individuals or patients (>18 years old) with acute leukemia who were on chemotherapy treatment or remission induction. No exclusion criteria were stated.	The authors discussed 2 categories of diet: low bacterial diet versus standard or normal hospital diet. Patients in both groups received antimicrobial prophylaxis.	Colonization of feces with *Candida* species or Gram-negative bacilli. Secondary findings were infection parameters and the total societal costs.	Data on stool colonization were very skewed and may not be analyzable. The study was financially supported by the Dutch Board and Profileringsfonds of the University Hospital Maastricht. The Medical Ethics Committee of the University Hospital Maastricht, The Netherlands approved the study protocol.
Ardakani et al [47]	A single-center, double-blind, randomized, placebo-controlled clinical trial conducted in Iran from April 2011 to August 2012	A total of 60 patients were enrolled (nonsmokers, aged ≥15 years, able to gargle the mouthwash solution, and capable of reading and communicating with staff). Exclusion criteria were no cooperation during the study, allergic reactions to herbal mouthwash, and failure to adhere to the oral health protocol due to any changes of their health condition.	A herbal mouthwash containing 1% dried extract of *Matricaria recutita*, 1% peppermint oil, and 99% ethanol. By contrast, the placebo mouthwash had similar taste, smell, and color, but contained 0.02% edible red color, 0.5% chlorophyllin color, 13% ethanol, and 71.5% distilled sterile water.	Development of oral mucositis assessed using the NCI-CTC^c^, with an evaluation of its duration, which was assessed by the number of days with the infection.	The study was registered with the Iranian Registry of Clinical Trials and approved by the Ethical Committee of Shahid Beheshti University of Medical Sciences.

^a^FFP2: filtering face piece.

^b^CVC: central venous catheter.

^c^NCI-CTC: National Cancer Institute-Common Terminology Criteria for Adverse Events (formerly Common Toxicity Criteria).

### Excluded Studies

We excluded 20 out of the short-listed 26 studies [48-67] as depicted in Multimedia Appendix 5. The studies were excluded due to the following reasons:

- Inappropriate study design: 5 nonrandomized comparative studies [50,53,55,58,64], 3 cohort studies [54,59,67], 3 case-control studies [51,52,56], 2 cross-sectional studies [62,65], 1 before-and-after study [63], 1 crossover study [49], and 1 mixed method study [61].
- Noninterest population: 2 studies [60,66].
- Nonrelevant intervention: 1 study [57].
- Nonrelevant outcome: 1 study [48].

We did not identify any on-going study for this review.

### Risk of Bias of the Included Studies

Overall, the risk-of-bias profile of the included studies varied, with insufficient information in most studies to enable a meaningful assessment of the risk-of-selection bias, and the high risk of performance bias in half of the included studies was due to a lack of blinding of individuals and personnel. The distribution of risk of bias in different aspects for the included studies are shown in Figures 2 and 3 and Multimedia Appendix 3.

### Effects of Interventions

#### Overview

All data presented were extracted from published reports. There were 8 comparisons included in the analysis, each only represented by a single study. The summarized analysis for the comparisons is tabulated in Multimedia Appendix 6. In the following sections, findings of the analysis are reported according to the comparison.

#### Comparison 1: Chlorhexidine and Nystatin Versus Saline Mouth Rinse

A single study was included [43], with 52 patients analyzed under this comparison out of the total of 99 patients from all 4 arms.

Only chemotherapy-related oral mucosal adverse effects were assessed in the study included [43]. However, there was no clear difference in mucositis score (grade 0-3, with higher scores indicating a more severe mucositis) between patients who received the chlorhexidine-nystatin mouth rinse and those who used the saline mouth rinse (MD 0.96, 95% CI –0.09 to 2.01; number of patients=52; analysis 1.1: quality of evidence was very low for both findings, reduced 1 level on the basis of risk of bias and another 2 levels due to severe serious concerns on imprecision). There was no clear difference in the average oral mucosal ulcer size between patients who received the chlorhexidine-nystatin mouth rinse and those who received the saline mouth rinse (MD 1.65 mm, 95% CI –7.48 to 10.78; number of patients=52; analysis 1.2: quality of evidence was very low for both outcomes, downgraded 1 level on the basis of risk of bias and another 2 levels due to severe serious concerns on imprecision). See also Multimedia Appendix 6 for the analysis.

#### Comparison 2: Chlorhexidine Versus Saline Mouth Rinse

A single study [43] was included, with 36 patients analyzed under this comparison out of the total of 99 patients from all 4 arms.

Only chemotherapy-related oral mucosal adverse effects were assessed in the included study [43]. However, there was no clear difference in mucositis score (grade 0-3, with higher scores indicating a more severe mucositis) between patients who received the chlorhexidine mouth rinse and patients who received the saline mouth rinse (MD 0.56, 95% CI –0.59 to 1.71; number of patients=36; analysis 2.1: quality of evidence was very low for both findings, reduced 1 level on the basis of risk of bias and another 2 levels due to severe serious concerns on imprecision). There was no clear difference in the oral mucosal ulcer size among patients who received the chlorhexidine mouth rinse and patients who received the saline mouth rinse (MD 2.17 mm, 95% CI –8.17 to 12.51; number of patients=36; analysis 2.2: quality of evidence was very low for both outcomes, reduced 1 level on the basis of risk of bias and another 2 levels due to severe serious concerns on imprecision).

#### Comparison 3: Nystatin Versus Saline Mouth Rinse

A single study [43] was included, with 34 patients analyzed under this comparison out of the total of 99 patients from all 4 arms.

Only chemotherapy-related oral mucosal adverse effects were assessed in the included study [43]. However, there was no clear difference in mucositis score (grade 0-3, with higher scores indicating a more severe mucositis) between patients who received the nystatin mouth rinse and patients who received the saline mouth rinse (MD 0.90, 95% CI –0.23 to 2.03; number of patients=34; analysis 3.1: quality of evidence was low, decreased 1 level on the basis of risk of bias and another 2 levels due to severe serious concerns on imprecision).

#### Comparison 4: Chlorhexidine Silver Sulfadiazine–Coated CVCs Versus Uncoated Catheters

A single study [42] of 184 catheters was included under this comparison. The study did not evaluate the risk of OI, but assessed CVC colonization, catheter-related bloodstream infection, and insertion site infection, which we classified for the purpose of this review as secondary findings. For catheter colonization the evidence showed that patients with cancer who received chlorhexidine silver sulfadiazine–coated CVCs appeared less likely to develop catheter colonization (RR 0.37, 95% CI 0.20 to 0.69; number of catheters=184; analysis 4.1: quality of evidence was moderate, downgraded 1 level based on indirectness of the outcome assessed).

For the association between catheters and bloodstream infections, the evidence showed no significant difference for the rate of catheter-related bloodstream infection between patients with cancer who received chlorhexidine silver sulfadiazine–coated CVCs and those who received standard, uncoated catheters (RR 0.45, 95% CI 0.12 to 1.68; number of catheters=184; analysis 4.2: quality of evidence was low, which was decreased 2 levels due to severe serious concerns on imprecision). For insertion site infection, there was no clear difference observed for the rate of insertion site infection between patients with cancer who received chlorhexidine silver sulfadiazine–coated CVCs and those who received standard, uncoated catheters (RR 0.94, 95% CI 0.66 to 1.33; number of catheters=184; analysis 4.3: quality of evidence observed was moderate, reduced 1 level because of serious concerns on imprecision).

#### Comparison 5: Well-Fitting Masks Versus No Mask

A single study [45] of 80 patients was included under this comparison. The study evaluated OI and all-cause mortality as the primary outcomes and mortality caused by OI as the secondary outcome.

In the included study [45], aspergillosis infection was assessed. The outcome was divided into possible, probable, or proven aspergillosis.

- For possible OIs, there was certainly no clear difference among patients who used a well-fitted mask versus those without a well-fitted mask (RR 0.48, 95% CI 0.09 to 2.45; number of patients=80; analysis 5.1.1: quality of evidence was very low, which was decreased by 3 levels due to indirectness and severe concerns on imprecision).
- For probable OIs, there was no clear difference among patients who used a well-fitted mask versus those without a well-fitted mask (RR 1.90, 95% CI 0.37 to 9.81; number of patients=80; analysis 5.1.2: quality of evidence was very low, which was reduced by 3 levels due to indirectness and serious concerns on imprecision).
- For proven OIs, there was no significant difference among patients who received a well-fitted mask versus those without a well-fitted mask (RR 0.95, 95% CI 0.14 to 6.43; number of patients=80; analysis 5.1.3: quality of evidence was very low, which was decreased by 3 levels due to indirectness and severe serious concerns on imprecision).
- For combined possible, probable, and proven OIs, there was no clear difference among patients who received a well-fitted mask versus those without a well-fitted mask (RR 0.95; 95% CI 0.40 to 2.29; number of patients = 80; analysis 5.1.4: very low quality of evidence, which was reduced by 3 levels for indirectness and severe concerns on imprecision).

The all-cause mortality provided by a single study [45], which was assessed clinically, showed no significant difference among patients with a well-fitted mask versus those without a well-fitted mask (RR 1.00, 95% CI 0.14 to 6.93; number of patients=160; analysis 5.2: reduced by 2 levels with low-quality evidence obtained for indirectness and severe concerns on imprecision). This study also provided evidence on mortality due to OI assessed clinically, in which no clear difference was observed for patients with and without well-fitted masks (RR 1.00, 95% CI 0.06 to 15.71; number of patients=160; analysis 5.3: quality of evidence was low, which was reduced by 2 levels due to indirectness and serious concerns on imprecision).

#### Comparison 6: Amine Fluoride-Stannous Fluoride Versus Sodium Fluoride Mouthwash

All-cause mortality provided by a single study [44], which was assessed clinically, showed no significant difference among patients who used the amine fluoride-stannous mouthwash versus those who used the sodium fluoride mouthwash (RR 0.67, 95% CI 0.11 to 3.88; number of patients=152; analysis 6.1: quality of evidence was low, reduced by 3 levels for indirectness and severe concerns on imprecision). This study also clinically assessed the combined adverse effect of stinging, discomfort in the mouth, teeth staining, nausea, and bad taste for patients who used the amine fluoride-stannous fluoride and sodium fluoride mouthwash, which showed a higher incidence of adverse effects for patients who used the latter (RR 9.33, 95% CI 1.34 to 64.89; number of patients=45; analysis 6.2.1: the very low-quality evidence reduced by 3 levels due to indirectness and serious concerns on imprecision).

#### Comparison 7: Low-Bacterial Diet Versus Normal Diet

One study [46] showed no clear difference in the impact of diet consumed (low bacterial vs normal) on OI (RR 0.2, 95% CI 0.01 to 3.70; number of patients=20; analysis 7.1.1: evidence with a very low quality was observed, which was reduced by 3 levels for indirectness and severe concerns on imprecision). For the OI (candidemia) assessed clinically and by laboratory reports, there was no clear difference among patients who consumed a low-bacterial diet versus those who consumed a normal diet (RR 1.00, 95% CI 0.07 to 13.87; number of patients=20; analysis 7.2: quality of evidence was very low, downgraded with 3 levels due to indirectness and serious concerns on imprecision).

#### Comparison 8: Herbal Mouthwash Versus Placebo Mouthwash

A single study [47] with 60 patients was analyzed under this comparison. The study evaluated oral mucositis. There was no significant difference between patients who received herbal and placebo mouthwash (RR 0.81, 95% CI 0.64 to 1.04; number of patients=60; analysis 8.1: evidence quality of evidence was moderate, downgraded by 1 level due to serious concerns on risk of incomplete outcome data bias).

## Discussion

### Summary of the Principal Findings

A total of 519 participants were evaluated from 6 included studies. Although this review included a small number of studies, it represented the best existing evidence that addressed the use of nondrug intervention for OIs. This review identified 4b major types of nondrug interventions for hematological cancer: (1) mouthwash that contained either chlorhexidine, nystatin, saline, amine fluoride-stannous fluoride, sodium fluoride, or herbal substances; (2) CVCs that were coated with chlorhexidine silver-sulfadiazine or uncoated; (3) use of well-fitted masks; and (4) diet consumed (either a low-bacterial diet or a normal diet). However, each type of intervention was represented by 1 small study. Overall, the use of chlorhexidine mouthwash alone or in combination with nystatin or nystatin mouthwash alone showed no clear difference for reductions in mucositis based on mucositis score and ulcer size [43]. A study by Laine et al [44] assessed the use of amine fluoride-stannous fluoride or sodium fluoride mouthwash. The study, however, did not show any clear difference in all-cause mortality and adverse effects such as discomfort in the mouth, teeth staining, unpleasant taste, and nausea. Maschmeyer et al [45] assessed the use of a well-fitted mask with no clear difference in OIs for all-cause mortality. Another study by Tiel et al [46] evaluated the use of a low bacteria diet versus a standard diet with no clear difference in the reduction of possible OIs or OIs assessed clinically. A study by Ardakani et al [47] that assessed the use of herbal mouthwash in preventing OIs showed no clear difference when compared with the placebo mouthwash group.

### Comparison With Prior Reviews or Studies

We found 2 published reviews that examined the effectiveness of nondrug interventions in preventing OIs among different populations. Helder et al [68] had assessed the effectiveness of 5 different nondrug interventions in preventing bloodstream infections among newborns admitted to a neonatal intensive care unit. The authors included 15 RCTs and found that proper CVC insertion and maintenance with a proper aseptic technique were the most effective interventions to prevent bloodstream infection in infants. The review differed from our review, as ours focused on evaluating the outcomes among adults with hematological malignancies. The other review by Wekesah et al [69] assessed the effectiveness of nondrug interventions in improving outcomes and quality of care among pregnant women in sub-Saharan Africa. The authors included 73 mixed design studies and identified many interventions for improving maternal health. The review differed in scope from our current review, as we only focused on the prevention of OIs among individuals with hematological cancers.

To the best of our knowledge, there is no systematic review that evaluated the use of nondrug interventions in preventing OIs among individuals with hematological cancer. The only available reviews that assessed interventions relevant to our review are those that evaluated antimicrobial-coated CVCs. One Cochrane systematic review and a related meta-analysis assessed the safety and effectiveness of antimicrobial catheters for patients in the intensive care unit, hematological and oncology unit (with all types of malignancies), and community settings [44,45], and reported that antimicrobial catheters in general reduced catheter colonization without clearly reducing catheter-related bloodstream infections, overall sepsis, and mortality rates. This is consistent with the finding of our single included study [42], which was also included in both reviews.

### Strengths

The review also has strengths. First, this is the first systematic review that evaluated the use of nondrug interventions for preventing OIs among patients with hematological cancers. We confirmed the effectiveness through the synthesis of evidence, and the result has significant clinical implication for both oncologists and patients. Second, we established strict inclusion and exclusion criteria, which resulted in a uniform data to be evaluated. We also included our assessment on study quality, which allows readers to judge the strength of evidence.

### Limitations

The review has a few limitations. First, despite our comprehensive search, we could find only very few studies related to OIs that are eligible to be included. This review also only focused on the effect of nondrug interventions for hematological cancers that limited the applicability of its results to other types of cancer or immunocompromised patients. Further, we might have missed relevant papers from smaller databases, especially those that are non-English. In addition, there might be publication bias that we were unable to rigorously evaluate as the number of included studies was too small. Besides, we only included RCTs in our review with a well-defined, relatively narrow set of patient population, and as such, serious or rare adverse events might not have been comprehensively captured.

### Conclusions

Overall, the quality of evidence presented in this review was very low. We are uncertain on the efficacy and safety of various types of mouthwash, coated CVCs, use of well-fitted masks, and low bacterial diet in major clinical findings such as OIs and related outcomes. Insufficient evidence exists on the effect of the nondrug intervention for preventing OIs in people with hematological cancers, and in people who are immunocompromised. This lack of evidence should be kept in mind when balancing the beneficial effects of nondrug interventions against the cost and feasibility of implementation in specific settings and against the potential for the development of OIs, and thus no firm conclusion can be made to inform practice. Therefore, further evidence is needed regarding the effect of nondrug interventions in patients with cancers, or in those who are immunocompromised.

## Appendix

Multimedia Appendix 1 Search strategy for MEDLINE.

Multimedia Appendix 2 Search strategy for CENTRAL (Cochrane Central Register of Controlled Trials).

Multimedia Appendix 3 Risk of bias for 6 included studies.

Multimedia Appendix 4 Summary of findings (SOF) tables.

Multimedia Appendix 5 Characteristics of the 20 excluded studies and their basis for exclusion.

Multimedia Appendix 6 Summary table of analysis for the 8 comparisons.

## Abbreviations

CENTRAL: Cochrane Central Register of Controlled Trials
CVC: central venous catheter
EHA: European Hematology Association
HEPA: high-efficiency particulate absorption
ICTRP: International Clinical Trials Registry Platform
MD: mean difference
MeSH: Medical Subject Heading
OI: opportunistic infection
PPE: personal protective equipment
PRISMA: Preferred Reporting Items for Systematic Reviews and Meta-Analyses
RCT: randomized controlled trial
RR: risk ratio
SOF: summary of findings
VAHO: Virginia Association of Hematologists and Oncologists
WHO: World Health Organization

## Data Availability

The original contributions shown in the study are included in this systematic review or its multimedia appendices; further inquiries can be addressed to the corresponding author.
