# Supplementary material for: Nondrug Intervention for Opportunistic Infections in Individuals With Hematological Malignancy: Systematic Review
Source: Interact J Med Res. 2023 Mar 31;12:e43969. doi: 10.2196/43969 (PMC10132047; doi:10.2196/43969)
Supplement: Multimedia Appendix 1 [file ijmr_v12i1e43969_app1.docx]

Multimedia Appendix 1

Title

**Nondrug Intervention for Opportunistic Infections in Individuals with Hematological Malignancy: Systematic Review**

Search Strategy for MEDLINE

| **#** | *Searches* |
| --- | --- |
| 1 | HEMATOLOGIC DISEASES/ |
| 2 | exp HEMATOLOGIC NEOPLASMS/ |
| 3 | (hematolog$ adj1 malignan$).tw,kf,ot. |
| 4 | (hematolog$ adj1 neoplas$).tw,kf,ot. |
| 5 | (haematolog$ adj1 malignan$).tw,kf,ot. |
| 6 | (haematolog$ adj1 neoplas$).tw,kf,ot. |
| 7 | exp BONE MARROW DISEASES/ |
| 8 | exp LYMPHOMA/ |
| 9 | exp LEUKEMIA/ |
| 10 | hodgkin$.tw,kf,ot. |
| 11 | lymphogranulomato$.tw,kf,ot. |
| 12 | lymphom$.tw,kf,ot. |
| 13 | histiocy$.tw,kf,ot. |
| 14 | granulom$.tw,kf,ot. |
| 15 | non-hodgkin$.tw,kf,ot. |
| 16 | nonhodgkin$.tw,kf,ot. |
| 17 | reticulosis.tw,kf,ot. |
| 18 | reticulosarcom$.tw,kf,ot. |
| 19 | (burkitt$ adj (lymph$ or tumo?r$)).tw,kf,ot. |
| 20 | lymphosarcom$.tw,kf,ot. |
| 21 | brill-symmer$.tw,kf,ot. |
| 22 | plasm##ytom$.tw,kf,ot. |
| 23 | myelom$.tw,kf,ot. |
| 24 | sezary.tw,kf,ot. |
| 25 | leuk?em$.tw,kf,ot. |
| 26 | myelodysplas$.tw,kf,ot. |
| 27 | aplast$ an?em$.tw,kf,ot. |
| 28 | or/1-27 |
| 29 | face masks/ |
| 30 | exp MASKS/ |
| 31 | (facemask$ or mask$).tw,kf,ot. |
| 32 | (facemask$ or face mask$).tw,kf,ot. |
| 33 | GLOVES, PROTECTIVE/ |
| 34 | GLOVES, SURGICAL/ |
| 35 | glove$.tw,kf,ot. |
| 36 | exp MOUTHWASHES/ |
| 37 | (rinse$ or rinsing or mouthrins$ or gargl$ or spray$ or tonsil$).tw,kf,ot. |
| 38 | DIET/ |
| 39 | diet&.tw,kf,ot. |
| 40 | AIR FILTERS/ |
| 41 | purifier$ air$.tw,kf,ot. |
| 42 | (air$ adj3 (filter$ or filtrat$)).tw,kf,ot. |
| 43 | exp DISINFECTANTS/ |
| 44 | (disinfectant$ or biocide$).tw,kf,ot. |
| 45 | or/29-44 |
| 46 | 28 and 45 |
| 47 | randomized controlled trial.pt. |
| 48 | controlled clinical trial.pt. |
| 49 | randomi$ed.ab. |
| 50 | placebo.ab |
| 51 | drug therapy.fs. |
| 52 | randomly.ab. |
| 53 | trial.ab. |
| 54 | groups.ab. |
| 55 | or/47-54 |
| 56 | humans.sh. |
| 57 | 55 and 56 |
| 58 | 46 and 57 |
