# Supplementary material for: Nondrug Intervention for Opportunistic Infections in Individuals With Hematological Malignancy: Systematic Review
Source: Interact J Med Res. 2023 Mar 31;12:e43969. doi: 10.2196/43969 (PMC10132047; doi:10.2196/43969)
Supplement: Multimedia Appendix 2 [file ijmr_v12i1e43969_app2.docx]

Multimedia Appendix 2

Title

**Nondrug Intervention for Opportunistic Infections in Individuals with Hematological Malignancy: Systematic Review**

Search Strategy for Cochrane Central Register of Controlled Trials (CENTRAL)

| #1 | Haematological malignancies:ti,ab,kw |
| --- | --- |
| #2 | MeSH descriptor: [Hematologic Neoplasms] explode all trees |
| #3 | cancer*:ti,ab,kw |
| #4 | oncolog*:ti,ab,kw |
| #5 | leukaemia*:ti,ab,kw |
| #6 | lymphoma*:ti,ab,kw |
| #7 | Non pharmacologic*:ti,ab,kw |
| #8 | face mask:ti,ab,kw |
| #9 | MeSH descriptor:[masks] explode all trees |
| #10 | glove*:ti,ab,kw |
| #11 | MeSH descriptor: [Gloves, Protective] explode all trees |
| #12 | mouthwash*:ti,ab,kw |
| #13 | MeSH descriptor: [Mouthwashes] explode all trees |
| #14 | diet:ti,ab,kw |
| #15 | MeSH descriptor: [Diet] explode all trees |
| #16 | disinfectant*:ti,ab,kw |
| #17 | MeSH descriptor: [Disinfectants] explode all trees |
| #18 | Air filter*:ti,ab,kw |
| #19 | MeSH descriptor: [Air filters] explode all trees |
| #20 | “positive pressure”:ti,ab,kw |
| #21 | (#1 OR #2 OR #3 OR #4 OR #5 OR #6) |
| #22 | (#7 OR #8 OR #9 OR #10 OR #11 OR #12 OR #13 OR #14 OR #15 OR #16 OR #17 OR #18 OR #19 OR #20) |
| #23 | (#21 AND #22) in Trials |
