# Supplementary material for: Nondrug Intervention for Opportunistic Infections in Individuals With Hematological Malignancy: Systematic Review
Source: Interact J Med Res. 2023 Mar 31;12:e43969. doi: 10.2196/43969 (PMC10132047; doi:10.2196/43969)
Supplement: Multimedia Appendix 3 [file ijmr_v12i1e43969_app3.docx]

Multimedia Appendix 3

Title

**Nondrug Intervention for Opportunistic Infections in Individuals with Hematological Malignancy: Systematic Review**

Risk of Bias for 6 Included Studies

| *Bias* | |  | *Study Author* | | | | | |
| --- | --- | --- | --- | --- | --- | --- | --- | --- |
|  |  | *Epstein 1992* (43) | | *Laine 1993* (44) | *Maschmeyer 2009* (45) | *Ostendorf 2005* (42) | *Tiel 2007* (46) | *Ardakani 2016* (47) |
| Random sequence generation (selection bias) | Authors’ judgement | High risk | | Unclear risk | Unclear risk | Unclear risk | Low risk | Low risk |
|  | Support for judgement | The authors reported patient’s allocation was made via randomization by sequential assignment in the individuals or patients on consecutive warded to the hospital. This is a form of alternation that we considered as the high risk of selection bias. | | The authors stated that participant allocation was made via randomization but there is no further detail was given on term of the method randomization was performed. | The authors reported the selection was based on randomization but no further details on how the randomization was made. A previous randomisation list for participating center was used. | Individuals or patients were distributed "through randomization ....". No further statements were reported on how random sequence was produced and how distribution was applied. | Patients were randomized into 2 categories based to the computerized randomized program. | The authors stated that participant allocation was made via randomisation by applying a consecutive sampling method, in which all eligible patients were randomly allocated in the study groups using the "RAND" command in Excel software. |
| Allocation concealment (selection bias) | Authors’ judgement | High risk | | Unclear risk | Unclear risk | Unclear risk | Unclear risk | Unclear risk |
|  | Support for judgement | As the study used some form of alternation, it considered as high risk of bias in allocation concealment. | | No relevant data or information was provided to allow a meaningful assessment of either random sequence was generated independently from allocation. | No information available regarding whether similar person involved in the randomization. | Individuals were allocated "through randomisation ...." No additional statements were created on how random sequence was created and how allocation was applied. | No information was provided. | No relevant information was provided to enable a meaningful assessment of whether random sequence was generated independently from allocation. |
| Participants blinding and personnel (performance bias) | Authors’ judgement | Low risk | | Unclear risk | High risk | Low risk | High risk | Low risk |
|  | Support for judgement | The types of mouth rinse were indistinguishable to patients. | | Although the information is not clearly reported by the authors, participants blinding and personnel was likely to have been achieved, as the authors stated that "the two mouthwashes were indistinguishable to patients". | It was not clear whether the patients and personnel were blinded to the allocation / intervention. However, blinding appeared was not possible as the intervention was obvious to the patients / participants. | Quote: "The two catheter types were indistinguishable to users and patients (double-blinded study design)." | The statement was not clear whether the patients and personnel were blinded to the allocation / intervention. However, blinding appeared not possible as this intervention was obvious to the patients. | Although not clearly stated by the authors, blinding of participants was likely to have been achieved, as the authors stated that the placebo mouthwash was similar in taste, smell and colour with the herbal mouthwash. |
| Blinding of outcome assessment (detection bias) | Authors’ judgement | Unclear risk | | Unclear risk | Unclear risk | Unclear risk | Unclear risk | Unclear risk |
|  | Support for judgement | No relevant information was provided by the author. | | The assessors of adverse effects, including stinging pain in the mouth and teeth, poor taste, and nausea in the participants themselves who were most likely to have been blinded. For the other major outcome of all-cause mortality, detection bias was unlikely whether the assessors were blinded, as this was an objective outcome. | There was no information on blinding of intervention was given by the researcher. | It was unclear whether the assessors of the microbiological outcomes were blinded to the status of the participants. | No information on blinding of intervention was given by the researcher. | No relevant information was provided by the authors. No information on outcome bias was presented. |
| Incomplete outcome data (attrition bias) | Authors’ judgement | Low risk | | Unclear risk | Low risk | High risk | Low risk | High risk |
|  | Support for judgement | There was 13% drop-out rate which was balanced between the groups. | | Out of 76, 34 patients (44.7%) did not complete the study. | Outcome data was completely described. All 80 patients randomized were analyzed during the study. | "Sixty-one catheters (24.9%) were excluded because of patient’s failure to notify the study team when the catheter was removed, or catheterization <24 hours.” The authors did not report the quantity of catheters by excluding from each group. Author reported that the baseline characteristics of the two groups after excluding the 61 catheters were same.  In view of the high exclusion level and the relatively low event rates, particularly for CRBSI and attributed death or mortality, we assessed the risk of attrition bias in this study as high. | Outcome data was completely described. All 20 patients were analyzed on risk of infection and cost analysis was performed during the study. | Out of 70, 10 patients (14.3%) did not complete the study due to patient's health status or lack of cooperation. |
| Selective reporting (reporting bias) | Authors’ judgement | High risk | | Unclear risk | Low risk | Low risk | Low risk | Low risk |
|  | Support for judgement | Data was clearly stated for primary outcomes. Out of 86 individuals before treatment, 62 (72%) had normal oral flora whereas 15 (18.8%) before treatment, were colonized in Candida species. About 7.1% patients’ organisms are not indigenous to the oral cavity were harbored. Furthermore, in the result the authors did not provide the standard deviation (SD) for each group. | | Two of the pre-specified outcomes, salivary microbial count and flow rate were not described in sufficient details to enable data extraction for meta-analysis (salivary flow rate was reported in summaries for men and women in both groups combined, and salivary microbial counts were presented in graphs). | Invasive aspergillosis was the primary outcome of the study. Data was clearly reported for primary & other important outcomes such as mortality. | Overall, the findings indicated in the Methods which is including colonization of catheter, CRBSI and attributed death or mortality and catheter-related local infection, that were reported in detail under results section. | Data was clearly reported for primary and other important outcomes such as infection by Candida sp. The cost analysis cost during hospitalization, after discharged and production costs during follow up. | Data was clearly reported for all outcomes. |
| Other bias | Authors’ judgement | Low risk | | Unclear risk | Low risk | Low risk | Unclear risk | Low risk |
|  | Support for judgement | No other bias was identified. | | There were marked difference between the two groups in their baseline characteristics, as the proportion of patients with non-Hodgkin's lymphoma was 23% in AmF+SnF group and 12% for NaF group, and the percentage of smokers was 33% in AmF+SnF group and 22% in NaF group. The responsible of these two factors in influencing the risk of opportunistic infection could not be discounted. | No other bias was determined. 3M Germany company supplied the mask for but not have additional responsible in the study. | None identified. | There was a baseline imbalance in the duration of chemotherapy cycles (number of days per treatment group) between the two groups. | No other bias was identified. |
