# Supplementary material for: Nondrug Intervention for Opportunistic Infections in Individuals With Hematological Malignancy: Systematic Review
Source: Interact J Med Res. 2023 Mar 31;12:e43969. doi: 10.2196/43969 (PMC10132047; doi:10.2196/43969)
Supplement: Multimedia Appendix 4 [file ijmr_v12i1e43969_app4.docx]

Multimedia Appendix 4

Title

**Nondrug Intervention for Opportunistic Infections in Individuals with Hematological Malignancy: Systematic Review**

# Summary of Findings (SOF) Tables

| *Table S1.* SOF for chlorhexidine-nystatin versus saline mouth rinse. | | | | | | | | | | |
| --- | --- | --- | --- | --- | --- | --- | --- | --- | --- | --- |
| **Patient or population** | | | | **:** | Patients with haematological malignancies | | | | | |
| **Setting** | | | | **:** | Haematology and Oncology Unit of a hospital | | | | | |
| **Intervention** | | | | **:** | Chlorhexidine-nystatin rinse | | | | | |
| **Comparison** | | | | **:** | Saline rinse | | | | | |
| **Outcomes** | | **Anticipated absolute effects* (95% CI)** | | | | | **Relative effect (95% CI)** | **№ of participants (studies)** | **Quality of the evidence (GRADE)** | **Comments** |
|  |  | **Risk with saline rinse** | | | | **Risk with chlorhexidine-nystatin rinse** |  |  |  |  |
| Opportunistic infection | |  | | | |  |  |  |  | Not assessed |
| All-cause mortality | |  | | | |  |  |  |  | Not assessed |
| Chemotherapy-related adverse effect: mucositis, assessed using mucositis score  (Scale 0 to 3, higher score indicating more severe mucositis) | | The mean mucositis score was 2.05 | | | | The mean mucositis score in the intervention group was 0.96 higher (0.09 lower to 2.01 higher) | - | 52  (1 RCT) | ⊕⊝⊝⊝  VERY LOW ^1 2^ |  |
| Chemotherapy-related adverse effect: oral mucosal ulceration (mean size in mm) | | The mean oral mucosal ulcer size was 5.94 mm | | | | The mean oral mucosal ulcer size in the intervention group was 1.65 mm larger (7.48 smaller to 10.78 larger) | - | 52  (1 RCT) | ⊕⊝⊝⊝  VERY LOW ^1 2^ |  |
| ***The** **risk in the intervention group** (and its 95% confidence interval) was based on the assumed risk in the comparison group and the **relative effect** of the intervention (and its 95% CI). | | | | | | | | | | |
| **CI:** Confidence interval; **RR:** Risk ratio; **OR:** Odds ratio; | | | | | | | | | | |
| **GRADE Working Group grades of evidence** | | | | | | | | | | |
| **High quality** | **:** | | We are very confident that the true effect lies close to that of the estimate of the effect. | | | | | | | |
| **Moderate quality** | **:** | | We are moderately confident in the effect estimate: The true effect is likely to be close to the estimate of the effect, but there is a possibility that it is substantially different. | | | | | | | |
| **Low quality** | **:** | | Our confidence in the effect estimate is limited: The true effect may be substantially different from the estimate of the effect. | | | | | | | |
| **Very low quality** | **:** | | We have very little confidence in the effect estimate: The true effect is likely to be substantially different from the estimate of effect. | | | | | | | |
| *Footnotes*  ^1^ There was a high risk of selection bias in the single included study. Quality of evidence downgraded   by one level.  ^2^ 95% CI was very wide, ranging from modestly lower to substantially higher score in relation to the   scale assessed, due to the small sample included in the analysis. Quality of evidence downgraded   by two levels due to very serious concern on imprecision, to the extent that the results were   practically non-informative with regards to the plausible range of effects. | | | | | | | | | | |

| *Table S2.* SOF for chlorhexidine versus saline mouth rinse. | | | | | | | | | | |
| --- | --- | --- | --- | --- | --- | --- | --- | --- | --- | --- |
| **Patient or population** | | | | **:** | Patients with haematological malignancies | | | | | |
| **Setting** | | | | **:** | Haematology and Oncology Unit of a hospital | | | | | |
| **Intervention** | | | | **:** | Chlorhexidine rinse | | | | | |
| **Comparison** | | | | **:** | Saline rinse | | | | | |
| **Outcomes** | | **Anticipated absolute effects* (95% CI)** | | | | | **Relative effect (95% CI)** | **№ of participants (studies)** | **Quality of the evidence (GRADE)** | **Comments** |
|  |  | **Risk with saline rinse** | | | | **Risk with chlorhexidine rinse** |  |  |  |  |
| Opportunistic infection | |  | | | |  |  |  |  | Not assessed |
| All-cause mortality | |  | | | |  |  |  |  | Not assessed |
| Chemotherapy-related adverse effect: mucositis, assessed using mucositis score  (Scale 0 to 3, higher score indicating more severe mucositis) | | The mean mucositis score was 2.05 | | | | The mean mucositis score in the intervention group was 0.56 higher (0.59 lower to 1.71 higher) | - | 36  (1 RCT) | ⊕⊝⊝⊝  VERY LOW ^1 2^ |  |
| Chemotherapy-related adverse effect: oral mucosal ulceration (mean size in mm) | | The mean oral mucosal ulcer size was 5.94 mm | | | | The mean oral mucosal ulcer size in the intervention group was 2.17 mm larger (8.17 smaller to 12.51 larger) | - | 36  (1 RCT) | ⊕⊝⊝⊝  VERY LOW ^1 2^ |  |
| ***The risk in the intervention group** (and its 95% confidence interval) was based on the assumed risk in the comparison group and the **relative effect** of the intervention (and its 95% CI). | | | | | | | | | | |
| **CI:** Confidence interval; **RR:** Risk ratio; **OR:** Odds ratio; | | | | | | | | | | |
| **GRADE Working Group grades of evidence** | | | | | | | | | | |
| **High quality** | **:** | | We are very confident that the true effect lies close to that of the estimate of the effect. | | | | | | | |
| **Moderate quality** | **:** | | We are moderately confident in the effect estimate: The true effect is likely to be close to the estimate of the effect, but there is a possibility that it is substantially different. | | | | | | | |
| **Low quality** | **:** | | Our confidence in the effect estimate is limited: The true effect may be substantially different from the estimate of the effect. | | | | | | | |
| **Very low quality** | **:** | | We have very little confidence in the effect estimate: The true effect is likely to be substantially different from the estimate of effect. | | | | | | | |
| *Footnotes*  ^1^ There was a high risk of selection bias in the single included study. Quality of evidence downgraded   by one level.  ^2^ 95% CI was very wide, ranging from modestly lower to substantially higher score in relation to the   scale assessed, due to the small sample included in the analysis. Quality of evidence downgraded   by two levels due to very serious concern on imprecision, to the extent that the results were   practically non-informative with regards to the plausible range of effects. | | | | | | | | | | |

| *Table S3.* SOF for nystatin versus saline mouth rinse. | | | | | | | | | | |
| --- | --- | --- | --- | --- | --- | --- | --- | --- | --- | --- |
| **Patient or population** | | | | **:** | Patients with haematological malignancies | | | | | |
| **Setting** | | | | **:** | Haematology and Oncology Unit of a hospital | | | | | |
| **Intervention** | | | | **:** | Nystatin rinse | | | | | |
| **Comparison** | | | | **:** | Saline rinse | | | | | |
| **Outcomes** | | **Anticipated absolute effects* (95% CI)** | | | | | **Relative effect (95% CI)** | **№ of participants (studies)** | **Quality of the evidence (GRADE)** | **Comments** |
|  |  | **Risk with saline rinse** | | | | **Risk with nystatin rinse** |  |  |  |  |
| Opportunistic infection | |  | | | |  |  |  |  | Not assessed |
| All-cause mortality | |  | | | |  |  |  |  | Not assessed |
| Chemotherapy-related adverse effect: mucositis, assessed using mucositis score  (Scale 0 to 3, higher score indicating more severe mucositis) | | The mean mucositis score was 2.05 | | | | The mean mucositis score in the intervention group was 0.9 higher (0.23 lower to 2.03 higher) | - | 34  (1 RCT) | ⊕⊝⊝⊝  VERY LOW ^1 2^ |  |
| ***The** **risk in the intervention group** (and its 95% confidence interval) was based on the assumed risk in the comparison group and the **relative effect** of the intervention (and its 95% CI). | | | | | | | | | | |
| **CI:** Confidence interval; **RR:** Risk ratio; **OR:** Odds ratio; | | | | | | | | | | |
| **GRADE Working Group grades of evidence** | | | | | | | | | | |
| **High quality** | **:** | | We are very confident that the true effect lies close to that of the estimate of the effect. | | | | | | | |
| **Moderate quality** | **:** | | We are moderately confident in the effect estimate: The true effect is likely to be close to the estimate of the effect, but there is a possibility that it is substantially different. | | | | | | | |
| **Low quality** | **:** | | Our confidence in the effect estimate is limited: The true effect may be substantially different from the estimate of the effect. | | | | | | | |
| **Very low quality** | **:** | | We have very little confidence in the effect estimate: The true effect is likely to be substantially different from the estimate of effect. | | | | | | | |
| *Footnotes*  ^1^ There was a high risk of selection bias in the single included study. Quality of evidence downgraded   by one level.  ^2^ 95% CI was very wide, ranging from modestly lower to substantially higher score in relation to the   scale assessed, due to the small sample included in the analysis. Quality of evidence downgraded   by two levels due to very serious concern on imprecision, to the extent that the results were   practically non-informative with regards to the plausible range of effects. | | | | | | | | | | |

| *Table S4.* SOF for chlorhexidine silver sulphadiazine coated central venous catheters versus uncoated catheters. | | | | | | | | | |
| --- | --- | --- | --- | --- | --- | --- | --- | --- | --- |
| **Patient or population** | | | | **:** | Patients with haematological malignancies | | | | |
| **Setting** | | | | **:** | Haematology and Oncology Unit of a hospital | | | | |
| **Intervention** | | | | **:** | Chlorhexidine silver-sulphadiazine coated central venous catheters | | | | |
| **Comparison** | | | | **:** | Uncoated catheters | | | | |
| **Outcomes** | **Anticipated absolute effects* (95% CI)** | | | | | **Relative effect (95% CI)** | **№ of participants (studies)** | **Quality of the evidence (GRADE)** | **Comments** |
|  | **Risk with uncoated catheters** | | | | **Risk with chlorhexidine** **silver-sulphadiazine coated central venous catheters** |  |  |  |  |
| Opportunistic infection |  | | | |  |  |  |  | Not assessed |
| All-cause mortality |  | | | |  |  |  |  | Not assessed |
| Catheter colonisation assessed with: Microbiological assessment follow up: range 7 days to 74 days | Study population | | | | | RR 0.37 (0.20 to 0.69) | 184  (1 RCT) | ⊕⊕⊕⊝ MODERATE ^1^ |  |
|  | 330 per 1,000 | | | | 122 per 1,000 (66 to 228) |  |  |  |  |
| Catheter related blood stream infection assessed with: clinical assessment follow up: range 7 days to 74 days | Study population | | | | | RR 0.45 (0.12 to 1.68) | 184  (1 RCT) | ⊕⊕⊝⊝ LOW ^2^ |  |
|  | 74 per 1,000 | | | | 34 per 1,000 (9 to 125) |  |  |  |  |
| Insertion site infection assessed with: clinical assessment follow up: range 7 days to 74 days | Study population | | | | | RR 0.94 (0.66 to 1.33) | 184  (1 RCT) | ⊕⊕⊕⊝ MODERATE ^3^ |  |
|  | 415 per 1,000 | | | | 390 per 1,000 (274 to 552) |  |  |  |  |
| ***The** **risk in the intervention group** (and its 95% confidence interval) was based on the assumed risk in the comparison group and the **relative effect** of the intervention (and its 95% CI). | | | | | | | | | |
| **CI:** Confidence interval; **RR:** Risk ratio; **OR:** Odds ratio; | | | | | | | | | |
| **GRADE Working Group grades of evidence** | | | | | | | | | |
| **High quality** | | **:** | We are very confident that the true effect lies close to that of the estimate of the effect. | | | | | | |
| **Moderate quality** | | **:** | We are moderately confident in the effect estimate: The true effect is likely to be close to the estimate of the effect, but there is a possibility that it is substantially different. | | | | | | |
| **Low quality** | | **:** | Our confidence in the effect estimate is limited: The true effect may be substantially different from the estimate of the effect. | | | | | | |
| **Very low quality** | | **:** | We have very little confidence in the effect estimate: The true effect is likely to be substantially different from the estimate of effect. | | | | | | |
| *Footnotes*  ^1^ Catheter colonisation was a surrogate for catheter related infection. Quality of evidence downgraded by   one level on the basis of indirectness.  ^2^ 95% CI was very wide, ranging from substantially lower risk to substantially higher risk. Quality of   evidence downgraded by two levels due to very serious concern with imprecision.  ^3^ 95% CI was wide, ranging from moderately lower risk to moderately higher risk. Quality of evidence   downgraded by one level due to serious concern with imprecision. | | | | | | | | | |

| *Table S5.* SOF for well-fitting mask versus no mask. | | | | | | | | | | |
| --- | --- | --- | --- | --- | --- | --- | --- | --- | --- | --- |
| **Patient or population** | | | | **:** | Preventing opportunistic infection in patients with haematological malignancies | | | | | |
| **Setting** | | | | **:** | Haematology and Oncology Unit of a hospital | | | | | |
| **Intervention** | | | | **:** | Well-fitting mask | | | | | |
| **Comparison** | | | | **:** | Without mask | | | | | |
| **Outcomes** | **Anticipated absolute effects* (95% CI)** | | | | | | **Relative effect (95% CI)** | **№ of participants (studies)** | **Quality of the evidence (GRADE)** | **Comments** |
|  | **Risk with without mask** | | | | | **Risk with well-fitting mask** |  |  |  |  |
| Opportunistic infection:  Fungal infection – Possible assessed microbiologicall follow up: range 4 days to 85 days | Study population | | | | | | RR 0.48 (0.09 to 2.45) | 80  (1 RCT) | ⊕⊝⊝⊝ VERY LOW ^1 2^ |  |
|  | 103 per 1,000 | | | | | 49 per 1,000 (9 to 251) |  |  |  |  |
| Opportunistic infection:  Fungal infection – Probable assessed microbiologicallyfollow up: range 4 days to 85 days | Study population | | | | | | RR 1.90 (0.37 to 9.81) | 80  (1 RCT) | ⊕⊝⊝⊝ VERY LOW ^1 2^ |  |
|  | 51 per 1,000 | | | | | 97 per 1,000 (19 to 503) |  |  |  |  |
| Opportunistic infection:  Fungal infection – Proven assessed microbiologicallyfollow up: range 4 days to 85 days | Study population | | | | | | RR 0.95 (0.14 to 6.43) | 80  (1 RCT) | ⊕⊝⊝⊝ VERY LOW ^1 2^ |  |
|  | 51 per 1,000 | | | | | 49 per 1,000 (7 to 330) |  |  |  |  |
| Opportunistic infection:  Fungal infection - Combined possible, probable and proven assessed microbiologicallyfollow up: range 4 days to 85 days | Study population | | | | | | RR 0.95 (0.40 to 2.29) | 80  (1 RCT) | ⊕⊝⊝⊝ VERY LOW ^1 2^ |  |
|  | 205 per 1,000 | | | | | 195 per 1,000 (82 to 470) |  |  |  |  |
| All-cause mortality assessed clinically follow up: range 4 days to 85 days | Study population | | | | | | RR 1.00 (0.14 to 6.293) | 160  (1 RCT) | ⊕⊕⊝⊝ LOW ^2^ |  |
|  | 25 per 1,000 | | | | | 25 per 1,000 (4 to 157) |  |  |  |  |
| Mortality due to opportunistic infection assessed clinically follow up: range 4 days to 85 days | Study population | | | | | | RR 1.00 (0.06 to 15.71) | 160  (1 RCT) | ⊕⊕⊝⊝ LOW ^2^ |  |
|  | 13 per 1,000 | | | | | 13 per 1,000 (1 to 171) |  |  |  |  |
| ***The** **risk in the intervention group** (and its 95% confidence interval) was based on the assumed risk in the comparison group and the **relative effect** of the intervention (and its 95% CI). | | | | | | | | | | |
| **CI:** Confidence interval; **RR:** Risk ratio; **OR:** Odds ratio; | | | | | | | | | | |
| **GRADE Working Group grades of evidence** | | | | | | | | | | |
| **High quality** | | **:** | We are very confident that the true effect lies close to that of the estimate of the effect. | | | | | | | |
| **Moderate quality** | | **:** | We are moderately confident in the effect estimate: The true effect is likely to be close to the estimate of the effect, but there is a possibility that it is substantially different. | | | | | | | |
| **Low quality** | | **:** | Our confidence in the effect estimate is limited: The true effect may be substantially different from the estimate of the effect. | | | | | | | |
| **Very low quality** | | **:** | We have very little confidence in the effect estimate: The true effect is likely to be substantially different from the estimate of effect. | | | | | | | |
| *Footnotes*  ^1^ This outcome was assessed using fungal culture which might not correlate well with clinical infection.   Quality of evidence downgraded by one level on the basis of indirectness.  ^2^ 95% CI was very wide, ranging from a substantial benefit to substantial harm for the intervention group   (well-fitting mask), due to the small sample included in the analysis. Quality of evidence downgraded by   two levels due to a very serious concern with imprecision, as the results are practically non-informative   with regards to the plausible range of effects. | | | | | | | | | | |

| *Table S6.* SOF for amine fluoride-stannous fluoride versus sodium fluoride mouthwash. | | | | | | | | | |
| --- | --- | --- | --- | --- | --- | --- | --- | --- | --- |
| **Patient or population** | | | | **:** | Preventing opportunistic infection in patients with haematological malignancies | | | | |
| **Setting** | | | | **:** | Haematology and Oncology Unit of a hospital | | | | |
| **Intervention** | | | | **:** | Amine fluoride-stannous fluoride mouthwash | | | | |
| **Comparison** | | | | **:** | Sodium fluoride mouthwash | | | | |
| **Outcomes** | **Anticipated absolute effects* (95% CI)** | | | | | **Relative effect (95% CI)** | **№ of participants (studies)** | **Quality of the evidence (GRADE)** | **Comments** |
|  | **Risk with sodium fluoride mouthwash** | | | | **Risk with amine fluoride-stannous fluoride mouthwash** |  |  |  |  |
| All-cause mortality, assessed clinically follow up: mean 12 months | Study population | | | | | RR 0.67 (0.11 to 3.88) | 152  (1 RCT) | ⊕⊝⊝⊝ VERY LOW ^1 2^ |  |
|  | 39 per 1,000 | | | | 26 per 1,000 (5 to 143) |  |  |  |  |
| Adverse effects - Combined (stinging pain in the mouth, staining of teeth, bad taste, nausea), assessed clinically follow up: mean 12 months | Study population | | | | | RR 9.33 (1.34 to 64.89) | 45  (1 RCT) | ⊕⊝⊝⊝ VERY LOW ^1 2^ |  |
|  | 56 per 1,000 | | | | 518 per 1,000 (74 to 1,000) |  |  |  |  |
| ***The** **risk in the intervention group** (and its 95% confidence interval) was based on the assumed risk in the comparison group and the **relative effect** of the intervention (and its 95% CI). | | | | | | | | | |
| **CI:** Confidence interval; **RR:** Risk ratio; **OR:** Odds ratio; | | | | | | | | | |
| **GRADE Working Group grades of evidence** | | | | | | | | | |
| **High quality** | | **:** | We are very confident that the true effect lies close to that of the estimate of the effect. | | | | | | |
| **Moderate quality** | | **:** | We are moderately confident in the effect estimate: The true effect is likely to be close to the estimate of the effect, but there is a possibility that it is substantially different. | | | | | | |
| **Low quality** | | **:** | Our confidence in the effect estimate is limited: The true effect may be substantially different from the estimate of the effect. | | | | | | |
| **Very low quality** | | **:** | We have very little confidence in the effect estimate: The true effect is likely to be substantially different from the estimate of effect. | | | | | | |
| *Footnotes*  ^1^ 34 out of 79 patients (43%) did not complete the trial, which by itself have seriously affected our confidence   on the results. Quality of evidence downgraded by one level due to high risk of attrition bias.  ^2^ 95% CI was very wide to the extent that the results are practically non-informative with regards to the   plausible range of effects. Quality of evidence downgraded by two levels due to very serious concern with   imprecision. | | | | | | | | | |

| *Table S7.* Low bacterial diet versus standard diet | | | | | | | | | |
| --- | --- | --- | --- | --- | --- | --- | --- | --- | --- |
| **Patient or population** | | | | **:** | Patients with haematological malignancies | | | | |
| **Setting** | | | | **:** | Haematology and Oncology Unit of a hospital | | | | |
| **Intervention** | | | | **:** | Low bacterial diet | | | | |
| **Comparison** | | | | **:** | Standard diet | | | | |
| **Outcomes** | **Anticipated absolute effects* (95% CI)** | | | | | **Relative effect (95% CI)** | **№ of participants (studies)** | **Quality of the evidence (GRADE)** | **Comments** |
|  | **Risk with standard diet** | | | | **Risk with low bacterial diet** |  |  |  |  |
| Opportunistic infection:  Invasive aspergillosis – Possible assessed with: clinical and laboratory means  follow up: range 406 days to 509 days | Study population | | | | | RR 0.2 (0.01 to 3.70) | 20  (1 RCT) | ⊕⊝⊝⊝ VERY LOW ^1 2^ | There was no event in the intervention (low-bacterial diet ) group while 2 out of 10 patients in the control (standard diet) group developed the event. |
|  | 200 per 1,000 | | | | 40 per 1,000 (2 to 740) |  |  |  |  |
| Opportunistic infection:  Invasive aspergillosis – Probable assessed with: clinical and means  follow up: range 406 days to 509 days | Study population | | | | | not estimable | 20  (1 RCT) |  | Not estimable as there were no events in either group. |
|  | 0 per 1,000 | | | | 0 per 1,000 (0 to 0) |  |  |  |  |
| Opportunistic infection:  Invasive aspergillosis – Proven  assessed with: clinical and laboratory means  follow up: range 406 days to 509 days | Study population | | | | | not estimable | 20  (1 RCT) |  | Not estimable as there were no events in either group. |
|  | 0 per 1,000 | | | | 0 per 1,000 (0 to 0) |  |  |  |  |
| Opportunistic infection:  Candidiemia  assessed with: clinical and laboratory means  follow up: range 406 days to 509 days | Study population | | | | | RR 1.00 (0.07 to 13.87) | 20  (1 RCT) | ⊕⊝⊝⊝ VERY LOW ^1 2^ |  |
|  | 100 per 1,000 | | | | 100 per 1,000 (7 to 1,000) |  |  |  |  |
| All-cause mortality |  | | | |  |  |  |  | Not assessed. |
| ***The** **risk in the intervention group** (and its 95% confidence interval) was based on the assumed risk in the comparison group and the **relative effect** of the intervention (and its 95% CI). | | | | | | | | | |
| **CI:** Confidence interval; **RR:** Risk ratio; **OR:** Odds ratio; | | | | | | | | | |
| **GRADE Working Group grades of evidence** | | | | | | | | | |
| **High quality** | | **:** | We are very confident that the true effect lies close to that of the estimate of the effect. | | | | | | |
| **Moderate quality** | | **:** | We are moderately confident in the effect estimate: The true effect is likely to be close to the estimate of the effect, but there is a possibility that it is substantially different. | | | | | | |
| **Low quality** | | **:** | Our confidence in the effect estimate is limited: The true effect may be substantially different from the estimate of the effect. | | | | | | |
| **Very low quality** | | **:** | We have very little confidence in the effect estimate: The true effect is likely to be substantially different from the estimate of effect. | | | | | | |
| *Footnotes*  ^1^ Non-blinding of participants to the diet that they received was likely to result in co-interventions that   might affect the outcome. Quality of evidence downgraded by one level due to serious concern on risk   of performance bias.  ^2^ 95% CI was very wide due to the very small number of events, which rendered the results non-  informative with regards to the plausible range of effects. Quality of evidence downgraded by two levels   due to very serious concerns with imprecision. | | | | | | | | | |

| *Table S8.* Herbal versus placebo mouthwash | | | | | | | | | |
| --- | --- | --- | --- | --- | --- | --- | --- | --- | --- |
| **Patient or population** | | | | **:** | Patients with haematological malignancies | | | | |
| **Setting** | | | | **:** | Bone Marrow Transplantation Center of Taleghani Hospital | | | | |
| **Intervention** | | | | **:** | Herbal mouthwash | | | | |
| **Comparison** | | | | **:** | Placebo mouthwash | | | | |
| **Outcomes** | **Anticipated absolute effects* (95% CI)** | | | | | **Relative effect (95% CI)** | **№ of participants (studies)** | **Quality of the evidence (GRADE)** | **Comments** |
|  | **Risk with standard diet** | | | | **Risk with low bacterial diet** |  |  |  |  |
| Opportunistic infection:  Oral mucositis | Study population | | | | | RR 0.81 (0.64 to 1.04) | 60  (1 RCT) | ⊕⊕⊕⊝ MODERATE ^1 2^ |  |
|  | 909 per 1,000 | | | | 736 per 1,000 (582 to 945) |  |  |  |  |
| ***The** **risk in the intervention group** (and its 95% confidence interval) was based on the assumed risk in the comparison group and the **relative effect** of the intervention (and its 95% CI). | | | | | | | | | |
| **CI:** Confidence interval; **RR:** Risk ratio; **OR:** Odds ratio; | | | | | | | | | |
| **GRADE Working Group grades of evidence** | | | | | | | | | |
| **High quality** | | **:** | We are very confident that the true effect lies close to that of the estimate of the effect. | | | | | | |
| **Moderate quality** | | **:** | We are moderately confident in the effect estimate: The true effect is likely to be close to the estimate of the effect, but there is a possibility that it is substantially different. | | | | | | |
| **Low quality** | | **:** | Our confidence in the effect estimate is limited: The true effect may be substantially different from the estimate of the effect. | | | | | | |
| **Very low quality** | | **:** | We have very little confidence in the effect estimate: The true effect is likely to be substantially different from the estimate of effect. | | | | | | |
| *Footnotes*  ^1^ The included study has high risk of bias in incomplete outcome data that was evaluated subjectively.  ^2^ The 95% CI ranges from an important increase to a clinically important increase contributed by a single   study. | | | | | | | | | |
