# Supplementary material for: Nondrug Intervention for Opportunistic Infections in Individuals With Hematological Malignancy: Systematic Review
Source: Interact J Med Res. 2023 Mar 31;12:e43969. doi: 10.2196/43969 (PMC10132047; doi:10.2196/43969)
Supplement: Multimedia Appendix 5 [file ijmr_v12i1e43969_app5.docx]

Multimedia Appendix 5

Title

**Nondrug Intervention for Opportunistic Infections in Individuals with Hematological Malignancy: Systematic Review**

Characteristics of 20 excluded studies and their basis for exclusion.

| *No.* | *Study author* | *Description for exclusion* | *Basis for exclusion* |
| --- | --- | --- | --- |
| 1 | Bauer 1994 (48) | This was a randomized controlled trial on cancer patients who received immuno-supportive diet. However, the study only evaluated physiological and biochemical outcomes with no clinical outcomes included. | Outcome |
| 2 | Bright-See 1985 (49) | This was a crossover study involving 28 participants divided into two groups who were followed until week six and crossed to another group for another six groups. | Study design |
| 3 | De Beule 1991 (50) | This study was not a randomized controlled trial. Patients were pre-selected earlier before the intervention. | Study design |
| 4 | Enig 1987 (51) | This was a case control study which accessed the meal preferences, the intake of nutrient, and nutritional status of the patients with cancer versus the healthy subject. | Study design |
| 5 | Franceschi 1989 (52) | This was a case control study. It was not the study design of interest. | Study design |
| 6 | Gardner 2008 (53) | This was a comparison study that evaluating the outcomes between the patients who consumed cooked food and uncooked food. It was not the study design of interest. | Study design |
| 7 | Hulshof 1987 (54) | This was an observational cohort study. it was not the study design of interest. | Study design |
| 8 | Jehn 1981 (55) | This was a non-randomized comparative study. | Study design |
| 9 | Kwiatkowski 1990 (56) | This was a case control study. It was not the study design of interest. | Study design |
| 10 | Levine 1975 (57) | The study assessed the effects of prophylactic antibiotics. It is not the intervention interest for this review. | Intervention |
| 11 | Levine 1982 (58) | This was a comparative study comparing the patients who receive adjuvant total parenteral nutrition and a standard diet. | Study design |
| 12 | Lindman 2013 (59) | This was a prospective study measuring the food caregivers that effect on the nutritional intake among warded haematological cancer individuals or patients. It was not the study design of interest. | Study design |
| 13 | McGaw 1985 (60) | Both groups of patients were treated with pharmacological chemotherapy regimen. The age range of the patients was from 17 until 54 years. Population of interest of our study was above 18 years old. | Population |
| 14 | McGrath 2002 (61) | The study was aimed to explore the experience in oncology/hematology treatment for both patients (using prospective study) and caregivers (using retrospective study) by using an explorative, descriptive, iterative and qualitative methodology. It is not the study design of interest. | Study design |
| 15 | Oren 2001 (62) | This was a cross sectional study and patients were treated with chemoprophylaxis. | Study design |
| 16 | Rico 1990 (63) | The study evaluates the effect of calcitonin on the bone lesions for the multiple myeloma. This study recruited 11 patients that treated with salmon calcitonin in nasal spray (200 IU) and 500 mg of elemental calcium/day for 3 months. This study was a pre-and post-treatment by using the comparison of biochemical and histomorphometric parameters. It was not the study design of interest. | Study design |
| 17 | Sugahara 2004 (64) | This was an "open trial with historical comparison" and not a randomized controlled trial study. It was not the study design of interest. | Study design |
| 18 | Trifilio 2012 (65) | This was a cross sectional comparison study to assess the impacts of diet in the general hospital and the neutropenic on the prevalence of the infections in the microbiologically. | Study design |
| 19 | Wahlin 1989 (66) | Children were recruited in this comparison study. Children are not the population of interest. | Population |
| 20 | Withington 1998 (67) | This was a cohort study which assessed the impact of intranasal amphotericin and HEPA filter. It was not the study design of interest. | Study design |
