# Supplementary material for: Nondrug Intervention for Opportunistic Infections in Individuals With Hematological Malignancy: Systematic Review
Source: Interact J Med Res. 2023 Mar 31;12:e43969. doi: 10.2196/43969 (PMC10132047; doi:10.2196/43969)
Supplement: Multimedia Appendix 6 [file ijmr_v12i1e43969_app6.docx]

Multimedia Appendix 6

Title

**Nondrug Intervention for Opportunistic Infections in Individuals with Hematological Malignancy: Systematic Review**

Summary table of analysis for the 8 comparisons

| **Comparison** | **Outcome or subgroup** | **Studies** | **Participants** | **Statistical method** | **Effect estimate** |
| --- | --- | --- | --- | --- | --- |
| Chlorhexidine and nystatin rinses versus saline rinse | 1.1 Chemotherapy-related adverse   effect: mucositis, assessed using   mucositis score | 1 | 52 | Mean difference (IV, Fixed, 95% CI) | 0.96 [-0.09, 2.01] |
|  | 1.2 Chemotherapy-related adverse   effect: oral mucosal ulceration   (mean size in mm) | 1 | 52 | Mean difference (IV, Fixed, 95% CI) | 1.65 [-7.48, 10.78] |
| **Chlorhexidine versus saline rinse** | 2.1 Chemotherapy-related adverse   effect: mucositis, assessed using   mucositis score | 1 | 36 | Mean difference (IV, Fixed, 95% CI) | 0.56 [-0.59, 1.71] |
|  | 2.2 Chemotherapy-related adverse   effect: oral mucosal ulceration   (mean size in mm) | 1 | 36 | Mean difference (IV, Fixed, 95% CI) | 2.17 [-8.17, 12.51] |
| **Nystatin rinse versus saline rinse** | 3.1 Chemotherapy-related adverse   effect: mucositis, assessed using   mucositis score | 1 | 34 | Mean difference (IV, Fixed, 95% CI) | 0.90 [-0.23, 2.03] |
| **Chlorhexidine Silver-sulfadiazine coated central venous catheters versus uncoated catheters** | 4.1 Catheter colonisation | 1 | 184 | Risk Ratio (M-H, Fixed, 95% CI) | 0.37 [0.20, 0.69] |
|  | 4.2 Catheter related blood stream   infection | 1 | 184 | Risk Ratio (M-H, Fixed, 95% CI) | 0.45 [0.12, 1.68] |
|  | 4.3 Insertion site infection | 1 | 184 | Risk Ratio (M-H, Fixed, 95% CI) | 0.94 [0.66, 1.33] |
| **Well-fitting mask versus no mask** | 5.1 Opportunistic infection: fungal   infection | 1 |  | Risk Ratio (M-H, Fixed, 95% CI) | Subtotals only |
|  | 5.1.1 Possible | 1 | 80 | Risk Ratio (M-H, Fixed, 95% CI) | 0.48 [0.09, 2.45] |
|  | 5.1.2 Probable | 1 | 80 | Risk Ratio (M-H, Fixed, 95% CI) | 1.90 [0.37, 9.81] |
|  | 5.1.3 Proven | 1 | 80 | Risk Ratio (M-H, Fixed, 95% CI) | 0.95 [0.14, 6.43] |
|  | 5.1.4 Combined | 1 | 80 | Risk Ratio (M-H, Fixed, 95% CI) | 0.95 [0.40, 2.29] |
|  | 5.2 All-cause mortality | 1 | 160 | Risk Ratio (M-H, Fixed, 95% CI) | 1.00 [0.14, 6.93] |
|  | 5.3 Mortality due to OI | 1 | 160 | Risk Ratio (M-H, Fixed, 95% CI) | 1.00 [0.06, 15.71] |
| **Amine fluoride stannous flouride versus sodium flouride mouthwash** | 6.1 All-cause mortality | 1 | 152 | Risk Ratio (M-H, Fixed, 95% CI) | 0.67 [0.11, 3.88] |
|  | 6.2 Adverse effects | 1 |  | Risk Ratio (M-H, Fixed, 95% CI) | Subtotals only |
|  | 6.2.1 Combined | 1 | 45 | Risk Ratio (M-H, Fixed, 95% CI) | 9.33 [1.34, 64.89] |
|  | 6.2.2 Stinging pain in mouth | 1 | 45 | Risk Ratio (M-H, Fixed, 95% CI) | 12.89 [0.80, 208.55] |
|  | 6.2.3 Staining of teeth | 1 | 45 | Risk Ratio (M-H, Fixed, 95% CI) | 1.33 [0.13, 13.64] |
|  | 6.2.4 Bad taste | 1 | 45 | Risk Ratio (M-H, Fixed, 95% CI) | 3.39 [0.17, 66.79] |
|  | 6.2.5 Nausea | 1 | 45 | Risk Ratio (M-H, Fixed, 95% CI) | 2.04 [0.09, 47.37] |
| **Low bacterial diet versus normal diet** | 7.1 Opportunistic infection: invasive   aspergillosis | 1 |  | Risk Ratio (M-H, Fixed, 95% CI) | Subtotals only |
|  | 7.1.1 Possible | 1 | 20 | Risk Ratio (M-H, Fixed, 95% CI) | 0.20 [0.01, 3.70] |
|  | 7.1.2 Probable | 1 | 20 | Risk Ratio (M-H, Fixed, 95% CI) | Not estimable |
|  | 7.1.3 Proven | 1 | 20 | Risk Ratio (M-H, Fixed, 95% CI) | Not estimable |
|  | 7.2 Opportunistic infection:   candidaemia | 1 | 20 | Risk Ratio (M-H, Fixed, 95% CI) | 1.00 [0.07, 13.87] |
| **Herbal vs placebo mouthwash** | 8.1 Opportunistic infection: oral   mucositis | 1 | 60 | Risk Ratio (M-H, Fixed, 95% CI) | 0.81 [0.64, 1.04] |
